# A novel biflavone from *Reineckia carnea* induces apoptosis of human renal cancer 786-O cells

**DOI:** 10.3389/fphar.2022.1053184

**Published:** 2022-12-01

**Authors:** Jianqiong Yang, Bang Xiao, Yamei Li, Xiaoxuan Liu, Minhong Zhang, Yaoling Luo, Biao Wang, Hai Liu

**Affiliations:** ^1^ The Clinical Medicine Research Center of the First Clinical Medical College, Gannan Medical University, Ganzhou, China; ^2^ School of Rehabilitation Medicine, Gannan Medical University, Ganzhou, China; ^3^ College of Pharmacy, Gannan Medical University, Ganzhou, China; ^4^ National Engineering Research Center for Modernization of Traditional Chinese Medicine-Hakka Medical Resources Branch, Gannan Medical University, Ganzhou, China

**Keywords:** Reineckia carnea, chemical constituents, reineckia-biflavone A, apoptosis, human renal cancer 786-O cells

## Abstract

Renal cell carcinoma (RCC) is a common malignant tumor of the urinary system, which is highly invasive, metastatic, and insensitive to radiotherapy and chemotherapy. Chinese herbal medicine has always been an important source of anti-tumor drug development. *Reineckia carnea* Kunth is a traditional herb commonly used by the Miao nationality in southwest China. In this study, the extract of *Reineckia carnea* was isolated and purified by reverse phase preparative chromatography and other chromatographic techniques. According to the physicochemical properties and spectral data, the structure of the compound was identified, and a novel biflavone compound named Reineckia-biflavone A (RFA) was obtained. The result of antiproliferative activity showed that RFA had cytotoxicity on 786-O cells with an IC_50_ value of 19.34 μmol/L. The results of CCK-8 and hemolysis assays showed that RFA was not significantly cytotoxic to both red blood cells (RBC) and peripheral blood mononuclear cells (PBMC). By Hoechst 33258 apoptosis staining, typical apoptotic morphology was observed under fluorescence microscope. RFA could induce the apoptosis of 786-O cells with the increase of apoptosis rate. The cell cycle tests showed that the cell proportion was obviously arrested in the S phase. At the same time, RFA could decrease the mitochondrial membrane potential and increase the intracellular free Ca^2+^ concentration. Western blot showed that the expression levels of pro-apoptotic proteins (Bax, Caspase-3, Cleaved Caspase-3, and Cytochrome c) in cells rose, while the expression level of anti-apoptotic proteins (Bcl-2) declined significantly. In conclusion, this study suggests that the RFA is a new biflavone determined by SciFinder retrieval. The apoptosis may be triggered by RFA through the mitochondrial pathway, which is mediated by up-regulating the intracellular calcium ion, down-regulating the mitochondrial membrane potential, and changing the apoptosis-related proteins.

## 1 Introduction

Renal cell carcinoma (RCC) is a common urological malignancy, a heterogeneous group of cancers caused by renal tubular epithelial cells, accounting for approximately 3% of all cancers (Pullen, 2021; Ljungberg et al., 2022). Clear cell renal cell carcinoma (cc-RCC) is the most common subgroup of RCC, accounting for approximately 75% of clinical cases of RCC (Hsieh et al., 2017). Although second only to prostate and bladder cancers in incidence, kidney cancer is considered the most lethal of all genitourinary tumors (Spadaccino et al., 2020). The global age-standardized incidence of RCC is estimated to be 4.4 cases per 100,000 population (Capitanio et al., 2019). According to statistics, there will be an estimated 431,288 new cases of renal cell carcinoma and 179,368 deaths worldwide in 2020 (Sung et al., 2021). So far, the specific mechanism of the occurrence and development of kidney cancer is still unclear, and the discovery of new biological markers is of great significance to the early diagnosis and prognosis of kidney cancer (Lai et al., 2017; Xue et al., 2019). Since kidney cancer is insensitive to radiotherapy, surgical resection remains the main treatment for kidney cancer, but surgical resection is only applicable to a small number of patients with early-stage tumors, while there is a high risk of recurrence and metastasis after surgery (Schrader et al., 2006; Li et al., 2018). Despite the development of immunotherapy in recent years, it still has some toxic side effects and most patients develop adaptive or intrinsic resistance mechanisms associated with disease progression due to a highly dynamic, adaptive, and heterogeneous tumor microenvironment (Duran et al., 2017; Heidegger et al., 2019). In addition, some commercial drugs used to treat kidney cancer can cause some adverse effects, such as hypertension, hand-foot syndrome, skin rash, and neutropenia (Barata and Rini, 2017). Therefore, new or even more effective therapeutic agents are urgently needed to be found. As a treasure of the Chinese nation, Traditional Chinese Medicine (TCM) is increasingly effective in cancer treatment and clinical practice, research has shown that TCM can activate the expression of oncogenes, promote apoptosis, and control the tumor microenvironment without serious side effects (Xiang et al., 2019).

*Reineckia carnea* Kunth is a perennial evergreen herb of the genus Reineckia in the Liliaceae family. It is a traditional herbal medicine commonly used by the Miao nationality in southwest China, with the effect of clearing the lung and relieving cough, cooling blood and stopping bleeding, detoxification, sore throat, *etc.* (Chen et al., 2011). It is commonly used in Miao folklore to treat cough, bronchitis, pneumonia, and other conditions, and its pharmacological effects are mainly reflected in anti-inflammatory and anti-tumor activities (Xing et al., 2018). Current domestic and foreign studies have reported the constituents of *Reineckia carnea*, including steroidal saponins (Xiang et al., 2020), flavonoids (Zhou et al., 2010), terpenoids (Yang et al., 2010), and lignans (Chen et al., 2011), of which the main constituents are steroidal saponins (Liu et al., 2012; Liu et al., 2015), and the most research has been conducted on such constituents, while less research has been reported on the other chemical constituents of the plant. In this experiment, a compound was isolated from the ethyl acetate extract fraction of 95% ethanol extract of *Reineckia carnea* and was identified as a novel biflavone compound named Reineckia-biflavone A (RFA). It was obtained from *Reineckia carnea* for the first time. According to current studies, biflavonoids have pharmacological activities such as antioxidant (Xiao et al., 2019), hypolipidemic (Ma et al., 2021), immunosuppressive (Ma et al., 2021), anti-inflammatory (Shim et al., 2018) and antitumor (Kang et al., 2021; Xie et al., 2022). Yu Ren et al. have reported a pro-apoptotic effect of Ginkgetin, a biflavonoid, on 786-O cells (Ren et al., 2016). In this study, human renal cancer 786-O cells were used as a cell model to study the anti-proliferation and apoptosis-inducing effects of RFA on renal carcinoma cells. Accordingly, the mechanism of apoptosis induced by RFA was discussed.

## 2 Materials and methods

### 2.1 Materials

The following apparatus were used during the experiment: INOVA-400 nuclear magnetic resonance instrument (Varian, United States , TMS as internal standard); HP-5973 mass spectrometer (Agilent, United States ); XRC-1 micro melting point tester; Rotary evaporator (Rotavapor R-220, Büchi Switzerland); Continuous spectrum multifunctional microplate reader (Varioskan Flash, Thermo Fisher, United States ); Fluorescence microscopy (IX 71, Olympus, Japan); Electronic balance (ME55, METTLER TOLEDO, Switzerland); CO_2_ incubator (Forma 311, Thermo, United States ); Flow cytometer (BD FACS Calibur, United States ); Molecular Imager ChemiDoc™ XRS imaging System (Bio-Rad, United States ).

200–300 mesh silica gel and GF254 silica gel plate were provided by Qingdao Marine Chemical Plant (Qingdao, China); Reversed phase chromatography silica gel C18 column was purchased from Merck in German; Sephadex LH-20 was purchased from GE in United States ; RPMI-1640 medium, DMEM medium, and fetal bovine serum were purchased from Gibco in United States ; Cell Counting Kit-8 was purchased from Gibco in United States ; dimethyl sulfoxide (DMSO) was purchased from Solarbio in China; Antibody (Cytochrome c and Cleaved Caspase-3)was purchased from Abcam in the United Kingdom; Antibody (Caspase-3, Bcl-2, and Bax) was purchased from Cell Signaling Technology in United States ; Human renal cancer 786-O cells and human embryonic kidney cells (HEK293T) were deposited and provided by the Clinical Medical Research Center of the First Affiliated Hospital of Gannan Medical University.

The whole plant of *Reineckia carnea* was collected from Anshun of Guizhou province in China. The herb was identified as *Reineckia carnea* (Andrews) Kunth, by Professor Qing-wen Sun of Guizhou University of Traditional Chinese Medicine. The voucher specimen (N0.20180701) was deposited in the herbarium of the Clinical Medical Research Center of the First Affiliated Hospital of Gannan Medical University.

### 2.2 Extraction and isolation

Hot reflux extraction with 95% ethanol from the dried whole herb of *Reineckia carnea* (10 kg) and the collected extract was concentrated under reduced pressure until there was no alcohol. The extract was extracted with petroleum ether and ethyl acetate after suspension with water. The ethyl acetate extract was mixed and subjected to silica gel column chromatography with gradient elution using CHCl_3_-MeOH (50∶1 → 2∶1). The elution portion of CHCl_3_-MeOH (20∶1) was collected and concentrated under reduced pressure to recover the solvent. The obtained concentrate was separated by ODS column chromatography, then gradient elution with MeOH-H_2_O (10∶90 → 90∶10). The portion of MeOH-H_2_O (40∶60 → 50∶50) was collected and concentrated under reduced pressure to recover the solvent and then separated by Sephadex LH-20 gel column chromatography. After collecting the MeOH-H_2_O (1∶1) part, 4 mg of red amorphous powder has been obtained.

### 2.3 Preparation of PBMC and RBC

Blood samples were obtained from healthy male volunteers. The study was approved by the institutional ethics committee of Gannan Medical University (NO: 2022366). Fresh blood samples were placed in tubes containing heparin anticoagulation and mixed with an equal volume of phosphate-buffered saline (PBS). A volume of Lymphocyte Separation Medium (Solarbio, Beijing, China) was added to the centrifuge tube in advance, followed by the slow addition of diluted blood samples along the tube wall. After centrifugation at 2000 rpm for 20 min, the PBMC layers and RBC layers were carefully sucked out.

### 2.4 Cell culture and cytotoxicity assay

786-O cells and HEK-293T cells were purchased from the cell bank of the Type Culture Collection Committee of the Chinese Academy of Sciences (Shanghai, China). 786-O cells and PMBC were routinely cultured in RPMI-1640 medium containing 10% fetal bovine serum (FBS), 1% penicillin, and 1% streptomycin. HEK-293T cells were cultured in DMEM medium containing 10% FBS, 1% penicillin, and 1% streptomycin. Both the cells were cultured in a humidified incubator with 5% CO_2_ at 37°C.

The cell proliferation was detected by CCK-8 assay. The 786-O cells in the logarithmic growth phase were prepared as single-cell suspensions and seeded in triplicate with 3,000 cells/well. After the cells adhered to the well, they were divided into four groups. The experimental group (RFA), the positive control group (paclitaxel), the negative control group (DMSO), and the blank group. RFA and paclitaxel were dissolved with DMSO to a certain concentration and subsequently diluted with medium to 2.5 μmol/L, 5 μmol/L, 10 μmol/L, 20 μmol/L, and 40 μmol/L. The cells were treated with RFA and paclitaxel at a concentration of (2.5 μmol/L, 5 μmol/L, 10 μmol/L, 20 μmol/L, 40 μmol/L) for 24, 48, 72 h, respectively. After that, the old medium was discarded, and 100 μL RPMI-1640 medium and 10 μL CCK-8 reaction solution was added to each well. After 2 h of incubation in a 37°C incubator, the absorbance of each well was detected at 450 nm by a continuous spectrum multifunctional microplate reader (Zhou et al., 2020). The same concentration gradients of RFA and paclitaxel were applied to PBMC for 24 h and HEK-293T for 72 h. The proliferative effects of RFA and paclitaxel on PBMC and HEK-293T were detected by the same method. The percentage of cell viability is calculated by the following formula: Cell viability (%) =(The OD value of the experimental group - The OD value of the blank group)/(The OD value of the negative control group - The OD value of the blank group)×100%.

### 2.5 Hemolytic assay

The Red Blood Cells were washed three times with PBS and then prepared into 2% Red Blood Cell Suspension with PBS. Different concentrations of test samples were mixed with 2.5 ml of 2% RBC solution to a final concentration of (2.5 μmol/L, 5 μmol/L, 10 μmol/L, 20 μmol/L, 40 μmol/L) and the total volume was 5 ml, incubated at 37 °C for 1 h, centrifuged at 2,500 rpm for 10 min, and the supernatant was taken. The absorbance was measured at 540 nm to determine the degree of hemolysis. Completely lysed blood is obtained by adding deionized water to a 2% RBC solution. Deionized water was used as a positive control and PBS was used as a negative control (Guo et al., 2013; Francis et al., 2015). The percentage of hemolysis is calculated by the following formula: Hemolysis (%) =(Sample OD-Negative control OD)/(Positive control OD-Negative control OD)×100%.

### 2.6 Cell apoptosis assay

Morphological changes of apoptosis were observed by using the Hoechst 33258 Detection Kit (KGA211; Nanjing KeyGen Biotech, China).786-O cells in good growth condition were inoculated in 6-well plates at a density of 2×10^5^ cells per well and treated with RFA at different concentrations (0 μmol/L, 10 μmol/L, 20 μmol/L, 40 μmol/L) for 24 h. Cells were washed twice with Buffer A (part of the Hoechst 33258 Detection Kit) and then fixed in methanol for 10 min at 4 °C. After fixation, the cells were washed twice with buffer A and incubated for 10 min at room temperature with Hoechst 33258 working solution obtained by diluting 10 times with Hoechst 33258 stock solution. The results were observed under fluorescence microscope and photographed.

The apoptotic rate of cells was detected by an annexin V-fluorescein isothiocyanate (FITC)/Propidium iodide (PI) apoptosis kit (556547; BD Pharmingen, United States ). 786-O cells were inoculated in 6-well plates at a density of 2×10^5^ cells per well and treated with RFA at different concentrations (0 μmol/L, 10 μmol/L, 20 μmol/L, 40 μmol/L) for 24 h. The cells of each well were separately collected and added to 5 µL of FITC Annexin V and 5 µL PI. Then the cells were gently mixed and reacted at room temperature for 15 min in dark. The rate of apoptotic cells (in the FITC+/PI- and FITC+/PI + quadrant) was measured by flow cytometry.

### 2.7 Cell cycle assay

786-O cells in the logarithmic growth phase were treated with RFA (0 μmol/L, 10 μmol/L, 20 μmol/L) for 24 h, respectively. The cells were fixed with 70% glacial ethanol at 4 °C for 24 h. Each group of cells was dyed in dark conditions with 300 μL propidium iodide (PI)/RNase stain buffer Staining Buffer (550825; BD Pharmingen, United States ) for 15 min and analyzed by flow cytometry.

### 2.8 Mitochondrial membrane potential (MMP) assay

Detection of mitochondrial membrane potential using the JC-10 Mitochondrial Membrane Potential Assay Kit (22801; AAT Bioquest, United States ). Cells were collected after trypsin digestion and added to 500 μL JC-10 dye solution. Incubated in dark for 20 min at 37 °C. The red fluorescence intensity and green fluorescence intensity of the cells were measured by flow cytometry. The mitochondrial membrane potential of the cells was expressed as the ratio of the intensity of red fluorescence to the intensity of green fluorescence.

### 2.9 Assessment of intracellular calcium concentration

The intracellular calcium concentration was measured with a Fluo-8^®^ Calcium Reagents and Screen Quest™ Fluo-8 NW Calcium Assay Kits (21081; AAT Bioquest, United States ). The 786-O cells which were treated with RFA (0 μmol/L, 10 μmol/L, 20 μmol/L, 40 μmol/L) for 24 h were collected, washed 3 times with D-Hanks solution, added Fluo-8/AM staining solution at a final concentration of 5 μmol/L, incubated for 30 min at 37°C in an incubator protected from light, and then washed 3 times by Hanks solution. Finally, the average fluorescence intensity of the cells was detected by flow cytometry.

### 2.10 Western blot assay

786-O cells in logarithmic phase were treated with 0 μmol/L, 10 μmol/L, 20 μmol/L, 40 μmol/L RFA for 24 h. The total proteins from each group were extracted by a RIPA buffer containing 1% PMSF (R0010; solarbio, China) and the total protein concentration was detected by a BCA reagent kit (P1511; Applygen, China). The proteins were separated by 12% SDS-PAGE and electrotransferred to the PVDF membrane. After sealing with 5% skimmed milk for 2 h, the membrane was incubated with specific primary antibody at 4 °C for 12 h, washed with TBST for three times, 5 min each time, and incubated with horseradish peroxidase-labeled secondary antibody for 1 h. Finally, the secondary antibody that did not bind to the primary antibody was washed by the above method and incubated with chemiluminescence solution to expose the strip in the dark room. The optical density of protein bands was analyzed by gel imaging analysis system.

### 2.11 Statistical analysis

Statistical analysis of the data was conducted using GraphPad Prism 8.0 software (San Diego 92108, CA, United States ). Data were presented in the form of mean ± SD (standard deviation). For the measurement data that conformed to the normal distribution, -tests were employed for comparison between the two groups, and one-factor ANOVA tests were used for comparison between the multiple groups. *P* < 0.05 was considered statistically significant.

## 3 Results

### 3.1 Structural identification of RFA

Red powders: [α]25.0 D: 15.6°(c 0.77, acetone); UV(acetone): λmax (log ε) = 327 nm (3.52); IR (KBr): max = 3,441, 2,923, 1,609, 1,552, 1,512, 1,421, 1,383, 1,271, 1,157, 1,109, 838 cm^−1^; Negative ESI-MS m/z (% rel. int.): 519.1 [M-H]-(100), Posotive ESI-MS m/z (% rel. int.): 521.2 [M + H]^+^(100); Its molecular formula was determined to be C_33_H_28_O_6_ based on the HR-ESI-MS m/z:521.1981 [M + H]^+^ (100) (calcd for C_33_H_28_O_6_, 521.1964). ^13^C-NMR and DEPT spectra showed that there are two methyl groups and one methoxy group, and there are two flavonoid C_6_-C_3_-C_6_ structures (ABC ring and A′B′C′ring).

**FIGURE 1:**
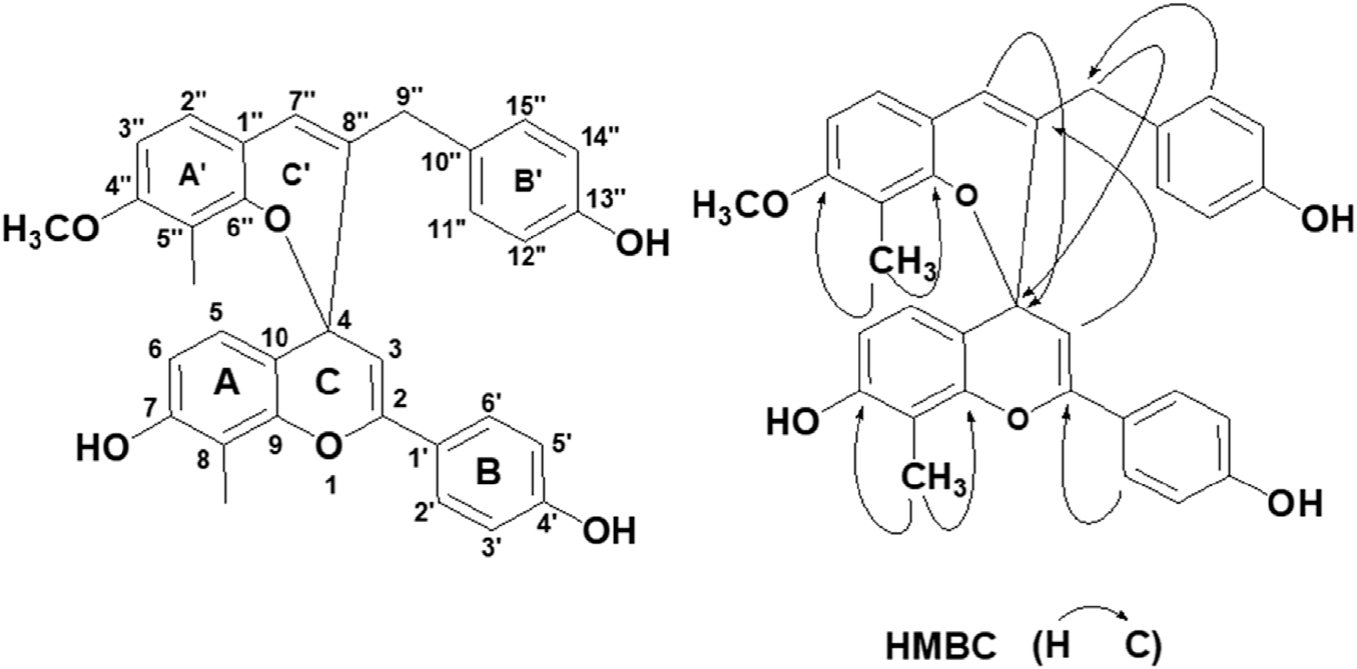
Structure of RFA and the key HMBC correlation.

In the ABC ring of the compound, δ_H_ 7.66 (2H, d, *J* = 8.0 Hz, H-2′, 6′) and 6.90 (2H, d, *J* = 8.0 Hz, H-3′,5′) indicated that the B ring was 1′, 4′substituted. In the ^1^H–^1^H COSY spectrum, the correlation of δ_H_ 7.20 (1H, d, *J* = 8.4 Hz, H-5) and 6.70 (1H, d, *J* = 8.4 Hz, H-6) indicated that the A ring had two adjacent hydrogens. In the HMBC spectrum, δ_H_ 2.32 (3H, s) was correlated with two oxygen-containing quaternary carbons [δc 150.2 (C-9), 156.7 (C-7)] (see Figure 1), indicating that monomethyl and −OH was located at eight and seven positions of A-ring, respectively. δ 5.71 (1H, s, H-3), 97.9 (C-3), and 149.7 (C-2) indicated the characteristic absorption peaks of flavonoids. H-2'. 6′ was also related to C-2 and H-5 was related to C-4 (77.8), which further verified that the C ring contained a pair of olefinic carbon (C-2, C-3) and an oxygenated carbon (C-4).

In the A′B′C′ring, the AA′BB′spin coupling system of δ_H_ 6.86 (2H, d, *J* = 7.6 Hz, H-11″, 15″) and 6.70 (2H, d, *J* = 7.6 Hz, H-12″,14″) indicates that the 13″position of B′ring has−OH substitution. The correlation of δ_H_ 6.76 (1H, d, *J* = 8.4 Hz, H-2″) and 6.42 (1H, d, *J* = 8.4 Hz, H-3″) in ^1^H-^1^HCOSY spectra shows that there are two adjacent hydrogens related to A ring. It can be seen that the side chain substitution structures of A′ring and B′ring are similar to those of A ring and B ring, respectively, and the only difference is that the 4″position of A′ring is replaced by an OCH_3_. This can be verified by the correlation between δ_H_ 3.74 (3H, s, -OMe) and δ_C_ (159.4, C-4″) in HMBC spectra. In addition, H-2″is related to C-7″, H-7″is related to C-9″, H-11″, H-15″is related to C-9″in HMBC spectra. It is found that A′ring and B′ring are connected by an allyl group, which contains a ethenyl [121.0 (C-7″), 138.3 (C-8″)] and a methylene [ 39.0 (C-9″)].

In the HMBC spectrum, the correlation between δ_H_ 5.92 (1H, s, H-7″) and δ_C_ 77.8 (C-4), δ_H_ 3.05 (2H, s, H-9″) and δ_C_ 77.8 (C-4), δ_H_ 5.71 (1H, s, H-3) and δc 138.3 (C−8″) (see Figure 1) indicates that C-4 and C-8″are connected. Therefore, ring C and ring C′ are formed through the central C-4 atom. Based on the above data, the compound was confirmed as a new compound and named Reineckia-biflavone A after searching similar literature and the SciFinder database. The structural formula is shown in Figure 1, and the ^1^H and ^13^C NMR data are shown in Table 1.

**TABLE 1:** ^1^H-NMR和^13^C-NMR data of RFA.

*No.*	*δ*_*H*_	*δ*_*C*_	*No.*	*δ*_*H*_	*δ*_*C*_
2		149.7	2″	6.76(1H,d,*J* = 8.4 Hz)	124.1
3	5.71(1H, s)	97.9	3″	6.42(1H,d,*J* = 8.4 Hz)	123.2
4		77.8	4″		159.4
5	7.20(1H, d, *J* = 8.4 Hz)	126.5	4″-OCH_3_	3.74(3H, s)	55.7
6	6.70(1H, d, *J* = 8.4 Hz)	112.1	5″		113.2
7		156.7	5″-CH_3_	1.80(3H, s)	8.4
8		111.7	6″		150.7
8-CH_3_	2.32(3H, s)	8.8	7″	5.92(1H, s)	121.0
9		150.2	8″		138.3
10		114.1	9″	3.05(2H, s)	39.0
1′		126.0	10″		130.0
2′,6′	7.66(2H, d, *J* = 8.0 Hz)	127.5	11″, 15″	6.86(2H,d,*J* = 7.6 Hz)	131.2
3′,5′	6.90(2H, d, *J* = 8.0 Hz)	116.2	12″, 14″	6.70(2H,d,*J* = 7.6 Hz)	115.8
4′		159.4	13″		156.6
1″		115.8			

### 3.2 Antiproliferative effect of RFA on 786-O cells

**FIGURE 2:**
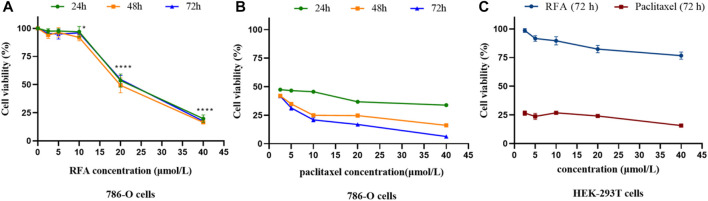
**(A,B)** The cell viability of 786-O cells under the treatment of RFA and paclitaxel at the different concentrations for 24, 48, and 72 h, respectively. **(C)**The cell viability of HEK-293T cells under the treatment of RFA and paclitaxel at different concentrations for 72 h, respectively. Data were expressed as mean ± SD. *p < 0.05, **p < 0.01, ****p < 0.0001, *versus* the untreated control group.

In this study, five concentration gradients of RFA (2.5 μmol/L, 5 μmol/L, 10 μmol/L, 20 μmol/L, 40 μmol/L) were selected to act on 786-O cells for 24, 48, and 72 h, and human embryonic kidney HEK-293T cells for 72 h, respectively. The inhibitory effect on cell proliferation was examined by performing the CCK-8 kit. The results showed that the proliferation inhibitory activity of RFA on 786-O cells increased with the increase of concentration in the range of 10 μmol/L, 20 μmol/L, and 40 μmol/L, showing in a dose-dependent but not time-dependent manner (Figure 2A). The calculated IC_50_ value of RFA on 786-O cells after 24 h was 19.34 μmol/L. Positive control (paclitaxel) showed time and dose dependence on 786-O cells (Figure 2B). At the same time, we found that the cell viability of HEK-293T cells treated by RFA for 72 h was almost unaffected compared to the control (Figure 2C).

### 3.3 Cytotoxicity of RFA on PBMC and RBC

**FIGURE 3:**
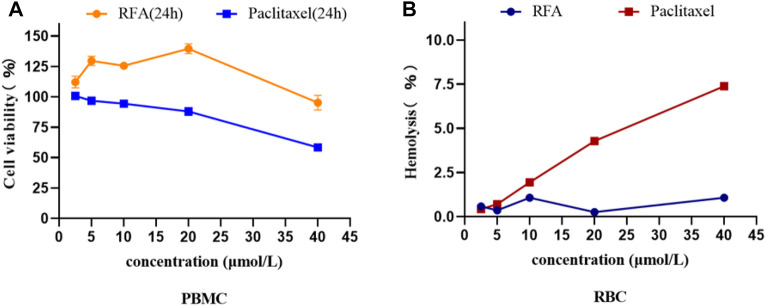
**(A)**The cell viability of PBMCs under the treatment of RFA and paclitaxel at different concentrations for 24 h, respectively. **(B)** Hemolysis rate of RBCs treated with different concentrations of RFA and paclitaxel. Data were expressed as mean ± SD.

The toxicity results of RFA on PBMC showed that the cytotoxic effect of RFA was significantly lower than that of paclitaxel at the same concentration, and it may have a pro-proliferative effect on PMBC in a certain concentration range (Figure 3A). In addition, the results of the hemolysis assay showed that is a process in which the integrity of the red blood cell membrane is cleaved or broken and hemoglobin is released. The effect of different concentrations (2.5 μmol/L, 5 μmol/L, 10 μmol/L, 20 μmol/L, 40 μmol/L) of RFA on hemolysis is shown in Figure 3B. RFA induced less than 2.5% hemolysis in intact erythrocytes, which was lower than that induced by paclitaxel.

### 3.4 Effect of RFA on apoptosis of 786-O cells

**FIGURE 4:**
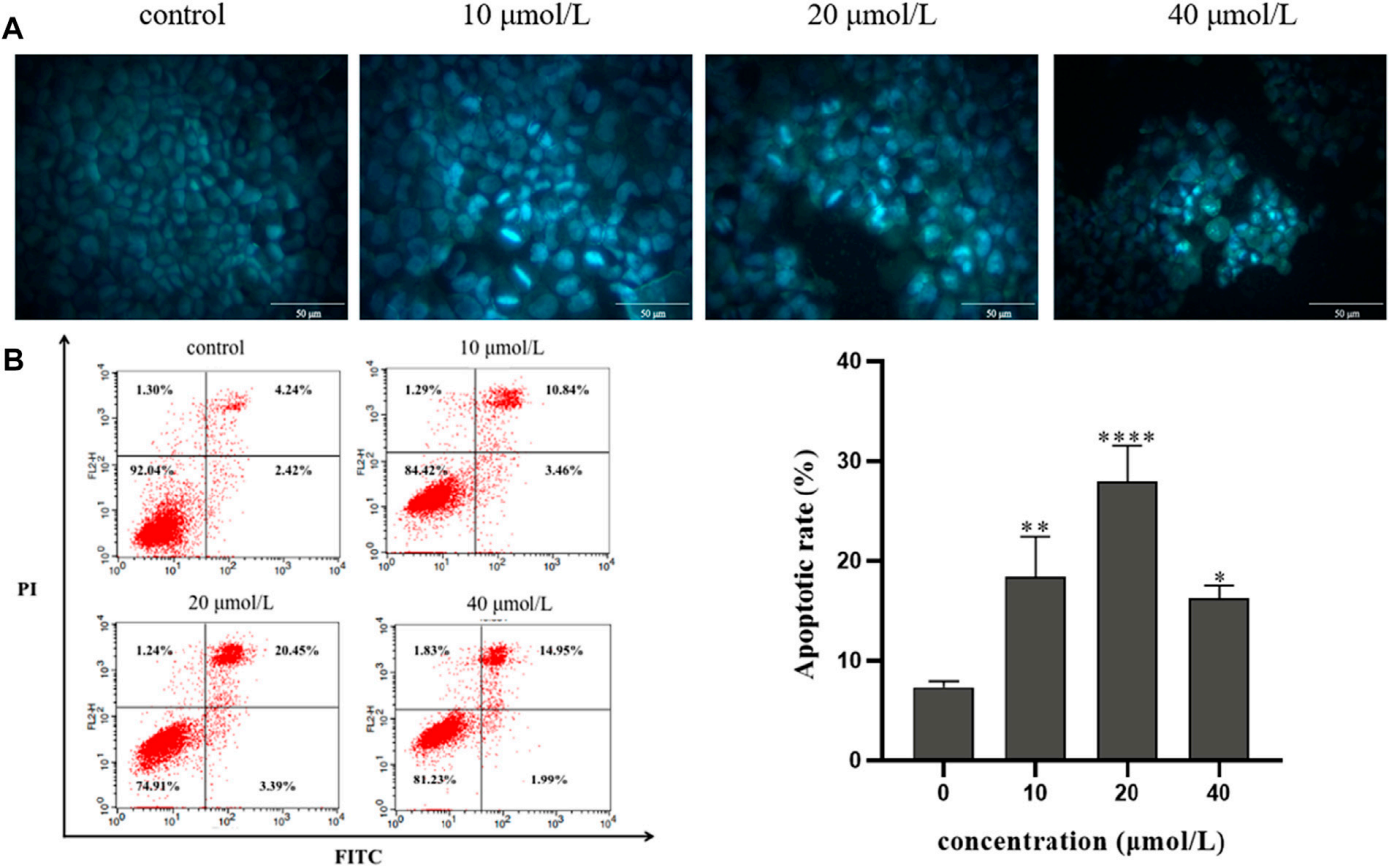
The effect of RFA on apoptosis of 786-O cells. **(A)** The morphology of apoptosis induced by RFA in 786-O cells was observed by fluorescence microscopy (400 ×). **(B)** The percentage of apoptosis induced by RFA was detected by Annexin V-FITC/PI double labeling. The apoptotic 786-O cells were in the FITC+/PI- and FITC+/PI + quadrant. Data were expressed as mean ± SD. *t*-test was used for the statistical analysis. *p < 0.05, **p < 0.01, ****p < 0.0001, *versus* the untreated control group.

To investigate the effect of RFA on apoptosis, 786-O cells were stained with Hoechst 33258 for 24 h after being treated with RFA at different concentrations (0 μmol/L, 10 μmol/L, 20 μmol/L, 40 μmol/L) and observed by fluorescence microscopy. The results showed that the distribution of nuclear chromatin in the normal control group was uniform, the cytoplasm was stretched, and the fluorescence intensity was low. Compared with the normal control group, The RFA-treated cells showed that the nuclei were solidified and sparsely grown under the microscope. At the same time, dense plaque-like chromatin was concentrated under the nuclear membrane, and the bright blue apoptotic body could be observed. The above are typical manifestations of apoptosis (Figure 4A).

Annexin V-FITC/PI double labeling method was used to detect the apoptosis rate by flow cytometry. After the 786-O cells were treated with RFA at different concentrations (0 μmol/L, 10 μmol/L, 20 μmol/L, 40 μmol/L) for 24 h, the apoptosis rate was observed. The results showed that compared with the normal control group, the apoptosis rate of 786-O cells in the experiment group was increased (Figure 4B). It is suggested that RFA could induce apoptosis of 786-O cells.

### 3.5 Effect of RFA on the cycle of 786-O cells

**FIGURE 5:**
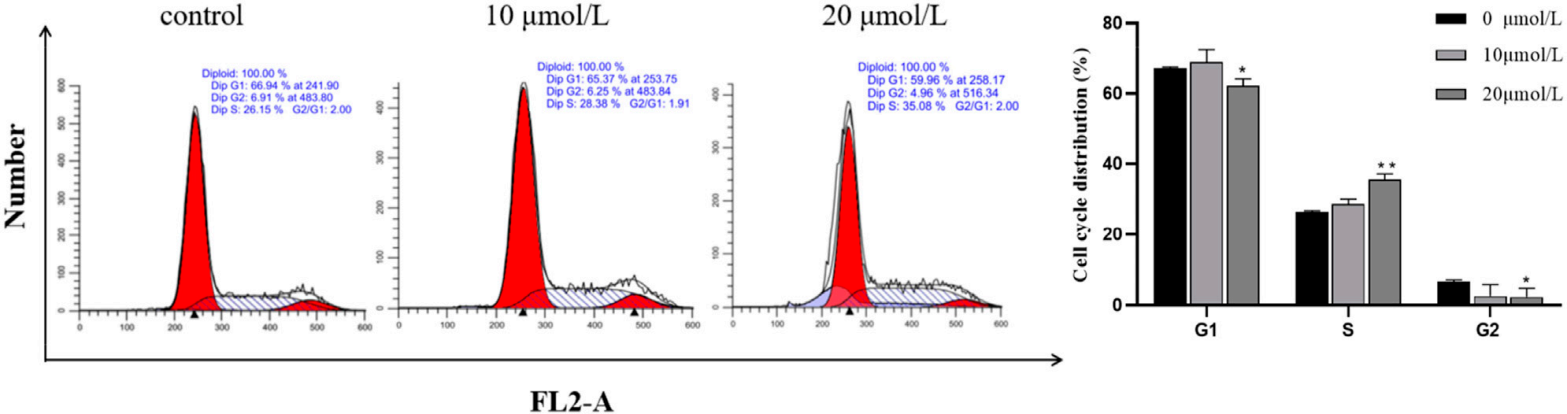
Effect of RFA on the cycle of 786-O cells. The proportion of cell numbers corresponding to the cell cycle (G1 phase, G2 phase, and S phase) in each group after treatment with different concentrations of RFA was demonstrated. Data were expressed as mean ± SD. The statistical methods were T-tests. *p < 0.05, **p < 0.01, *versus* the untreated control group.

After the RFA at different concentrations (0 μmol/L, 10 μmol/L, 20 μmol/L) was applied to 786-O cells for 24 h, the results showed that the cells in G1 and G2 phases decreased, and the cells in S phase increased. Combined with the results of the proliferation inhibition rate test of the above cells, it was indicated that the RFA may induce S cell cycle arrest and induce apoptosis in 786-O cells. (Figure 5).

### 3.6 Effect of RFA on the mitochondrial membrane potential of 786-O cells

**FIGURE 6:**
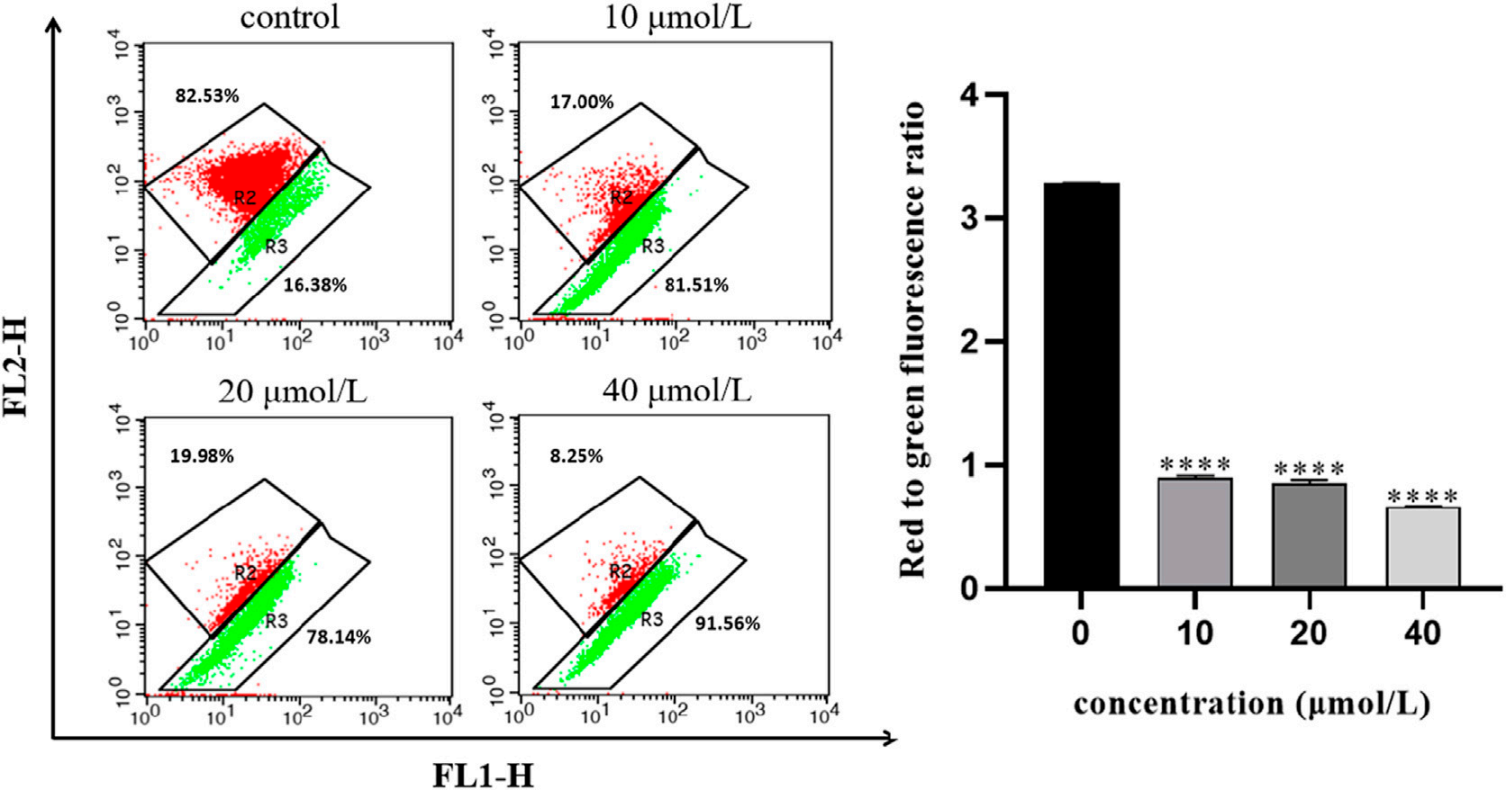
Effect of RFA on the mitochondrial membrane potential of 786-O cells. The mitochondrial membrane potential was presented as the ratio of the red fluorescence intensity to the green fluorescence intensity. Data were expressed as mean ± SD. *t*-test was used for the statistical analysis of the data. ****p < 0.0001, *versus* the untreated control group.

The opening of mitochondrial membrane permeable transport channels is the premise for Cytochrome c in mitochondria to be transported to the cytoplasm and is a necessary event to initiate apoptosis through the mitochondrial pathway. To investigate whether the apoptosis of 786-O cells induced by RFA affects the mitochondrial membrane potential, the effect of RFA on the mitochondrial membrane potential of 786-O cells was measured by JC-10 labeling. After different concentrations of RFA acted on 786-O cells for 24 h, compared with the normal control group, the red fluorescence changed to green fluorescence and the ratio of red fluorescence intensity to green fluorescence intensity decreased (Figure 6). The results showed that the mitochondrial membrane potential level of 786-O cells was obviously decreased after being treated with RFA.

### 3.7 Assessment of intracellular calcium concentration

**FIGURE 7:**
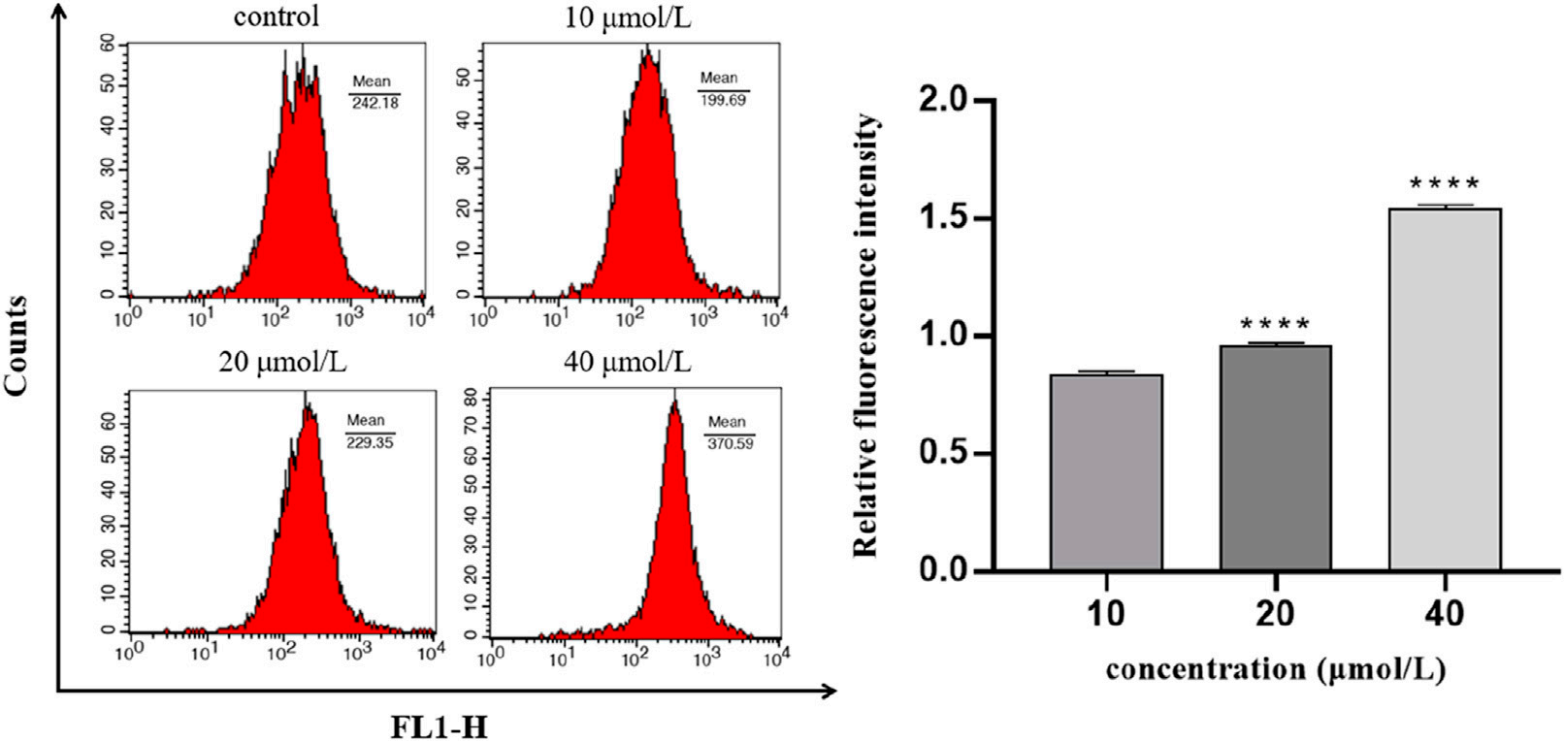
The concentration of intracellular calcium in 786-O cells induced by RFA. The relative level of intracellular calcium was shown as the ratio of the fluorescence intensity of the treated group to the blank group. Data were expressed as mean ± SD. *t*-test was used for the statistical analysis of results. ****p < 0.0001, *versus* the low concentration group (10 μmol/L).

To explore whether intracellular calcium signal is involved in the regulation of 786-O cell apoptosis induced by RFA, intracellular calcium concentration was determined in this experiment. The results showed that after the 786-O cells were treated with different concentrations of RFA for 24 h, the intracellular calcium ion level in each RFA treatment group increased with the increasing concentration (Figure 7). It indicated that intracellular calcium ion signal may be involved in the regulation of RFA-induced apoptosis of 786-O cells.

### 3.8 Effect of RFA on apoptosis-related protein expression in 786-O cells

**FIGURE 8:**
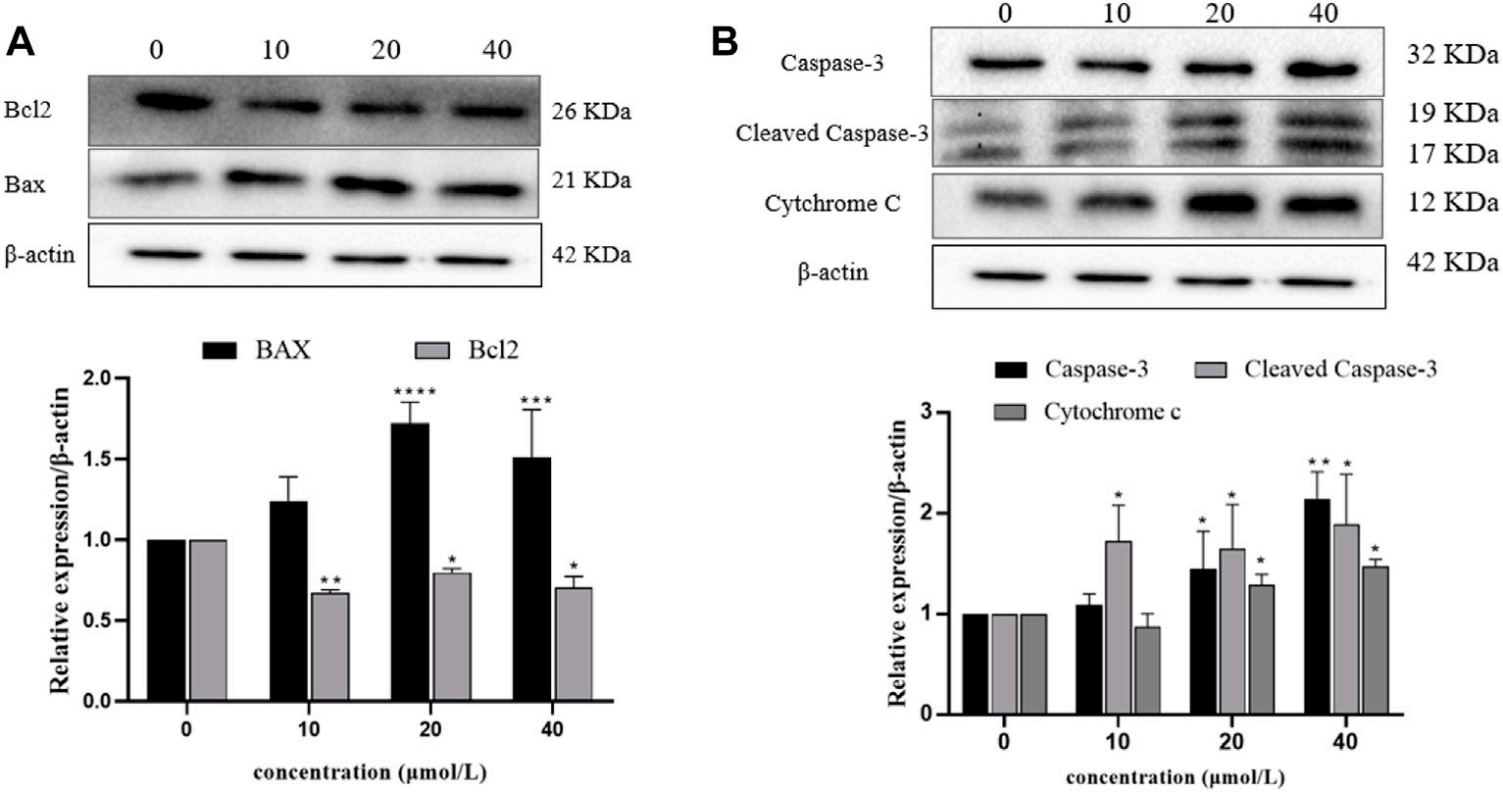
Expression levels of apoptosis-related proteins in 786-O cells after RFA treatment. Western blotting was used to determine. **(A)** The expression levels of Bax and Bcl-2 proteins. **(B)** The expression levels of Caspase-3, Cleaved Caspase-3, and Cytochrome c proteins. Data were expressed as mean ± SD. *t*-test was used for the statistical analysis. *p < 0.05, **p < 0.01, ***p < 0.001, ****p < 0.0001, *versus* the untreated control group.

In order to analyze the relative expression levels of apoptosis-related proteins (Bax, Bcl-2, Caspase-3, Cleaved Caspase-3, and Cytochrome c) in 786-O cells treated with RFA for 24 h, Western blot was used for determination. The results showed that compared with the negative control group, the expression levels of pro-apoptotic proteins (Bax, Caspase-3, Cleaved Caspase-3, and Cytochrome c) in the RFA treatment group were significantly increased, while the expression level of anti-apoptotic proteins (Bcl-2) was significantly decreased (Figure 8).

## 4 Discussion and conclusion

In this study, we firstly investigated the chemical composition of *Reineckia carnea*, from which a new biflavone compound, named Reineckia-biflavone A, was isolated and identified.

Then, we examined the cytotoxic activity of RFA on 786-O cells, and the results showed that the proliferation of 786-O cells was significantly inhibited by RFA in a dose-dependent manner. Although fewer adverse effects are the advantage of natural products, it is critical to assess the safety of newly discovered compounds using red blood cells and immune cells (Kundishora et al., 2020). The results clearly showed that RFA had no obvious cytotoxicity to PBMC, and may promote proliferation within a certain concentration range. Hemolysis was regarded as the red blood cell toxicity index, the red blood cell hemolysis test of RFA showed that the percentage of hemolysis was less than 2.5%, which met the recognized requirement that the hemolysis rate should not exceed 5% (Guo et al., 2013; Francis et al., 2015). Therefore, we preliminarily believe that RFA is non-toxic and safe. The experiment of cell cycle arrest was used to study the inhibition of the tumor cell growth and halt of cell cycle progression (Lin et al., 2018), we found that the cells in S-phase were increased and the cells in G1 and G2 phases were decreased. This indicates that RFA can induce 786-O cell cycle arrest in the S phase to inhibit cell proliferation.

Hoechst 33258 fluorescence staining experiment showed the typical apoptotic morphology phenomenon in RFA-treated 786-O cells such as nuclear condensation and chromatin condensation under a fluorescence microscope. Meanwhile, the results of flow cytometry showed that the increase of apoptosis rate depended on RFA concentration. Based on the above results, we confirmed the RFA has the potential to promote apoptosis in 786-O cells.

The development of apoptosis-targeted antitumor drugs has received much attention because apoptosis-induced cell death causes less inflammatory response (Jan and Chaudhry, 2019). Current studies suggest that apoptotic pathways include endogenous and exogenous pathways triggered by cellular stress, DNA damage, and immune surveillance mechanisms (Carneiro and El-Deiry, 2020). The exogenous apoptotic pathway, also known as the death receptor pathway, consists of the binding of TNF family ligands such as TNF to the death receptor on the cell membrane, and the apoptotic signal is transmitted to the cell, which then initiates the process of apoptosis (Pfeffer and Singh, 2018). The endogenous pathway, also known as the mitochondrial pathway, is induced by apoptosis-inducing factors, causing changes in mitochondrial membrane permeability and the release of pro-apoptotic proteins such as Cyt-c into the cytoplasm to trigger apoptosis (Lopez and Tait, 2015). In order to determine the type of apoptosis induced by RFA, we used JC-10 labeling to determine the effect of RFA on the mitochondrial membrane potential of 786-O cells, and the results showed that the level of mitochondrial membrane potential decreased in the RFA-treated cells. It is suggested that RFA may induce apoptosis through the mitochondrial pathway in 786-O cells.

An important part of the mitochondrial apoptosis pathway is the change of mitochondrial permeability, which is realized through the mitochondrial permeability transition pore (MPTP) (Šileikytė and Forte, 2019). It has been proved that Bcl-2 family proteins are important components of MPTP, mainly divided into anti-apoptotic (Bcl-2, Bcl-xl, *etc.*) and pro-apoptotic (Bax, Bcl-xs, *etc.*) (Patel et al., 2021). When the cells were stimulated by various apoptotic signals, the conformation of Bax protein changed, forming homodimers, stimulating the release of apoptotic factors such as Cyt-c into the cytoplasm, forming a polyplex with apoptotic protease activator-1, and promoting the self-activation of Caspase-9 precursor. The activated Caspase-9 then activates the downstream Caspase-3, resulting in apoptosis (Correia et al., 2015; Montero et al., 2020). Bcl-2 in the mitochondrial outer membrane can competitively bind to Bax protein, inhibit the formation of Bax homodimer, and inhibit the occurrence of apoptosis (Correia et al., 2015; Hu et al., 2020). It can be seen that the balance between pro-apoptotic proteins and anti-apoptotic proteins in normal cells is very important. When the balance between Bcl-2 and Bax is broken, the permeability of MPTP will be changed, leading to the release of proapoptotic factors such as Cyt-c (Chen and Lesnefsky, 2011; Izzo et al., 2016). Therefore, we examined the levels of mitochondrial pathway-related proteins, and the results showed that the expression levels of Bax, Cyt-c, Caspase-3, and Cleaved Caspase-3 were significantly increased and the expression level of the apoptosis-inhibiting protein Bcl-2 was significantly decreased in RFA-treated 786-O cells. It was further confirmed that apoptosis was induced through the mitochondrial pathway. Calcium ions are an important intracellular second messenger and are also involved in early apoptotic signaling. It has been reported that when the intracellular calcium ion concentration is too high, the mitochondria accumulate a large amount of calcium ions and become overloaded, the MPTP opens and the mitochondrial membrane potential changes, leading to Cyt-c release (De Marchi et al., 2014a; Dewangan et al., 2018a). In order to explore whether calcium ions participate in the RFA-induced apoptosis of 786-O cells, after Fluo-8/AM staining, the flow cytometry analysis showed that the increase in intracellular free calcium was dependent on the concentration of RFA, indicating that calcium ions may be involved in the regulation of RFA-induced apoptosis of 786-O cells.

In conclusion, RFA extracted from *Reineckia carnea*, a new biflavone with good biocompatibility, could inhibit the proliferation of 786-O cells *in vitro* and induce apoptosis through the mitochondrial pathway. The above tentatively confirmed that RFA has the effect of inducing apoptosis in kidney cancer cells, but the specific mechanism of action of RFA against renal cell carcinoma is not well understood and needs to be further explored. Meanwhile, whether RFA has other pharmacological effects is still unclear and needs to be further investigated.

## Data Availability

The original contributions presented in the study are included in the article/Supplementary Material, further inquiries can be directed to the corresponding author.

## References

[B1] Barata P. C. Rini B. I. (2017). Treatment of renal cell carcinoma: Current status and future directions. Ca. Cancer J. Clin. 67 (6), 507–524. 10.3322/caac.21411 28961310

[B2] Capitanio U. Bensalah K. Bex A. Boorjian S. A. Bray F. Coleman J. (2019). Epidemiology of renal cell carcinoma. Eur. Urol. 75 (1), 74–84. 10.1016/j.eururo.2018.08.036 30243799 PMC8397918

[B3] Carneiro B. A. El-Deiry W. S. (2020). Targeting apoptosis in cancer therapy. Nat. Rev. Clin. Oncol. 17 (7), 395–417. 10.1038/s41571-020-0341-y 32203277 PMC8211386

[B4] Chen L. L. Han N. Wang Y. C. Huang T. Xue R. Yin J. (2011). The chemical constituents from whole plant of Reineckia carnea( Andr. ) Kunth. J. Shenyang Pharm. Univ. 28 (11), 875–878. 10.14066/j.cnki.cn21-1349/r.2011.11.004

[B5] Chen Q. Lesnefsky E. J. (2011). Blockade of electron transport during ischemia preserves bcl-2 and inhibits opening of the mitochondrial permeability transition pore. FEBS Lett. 585 (6), 921–926. 10.1016/j.febslet.2011.02.029 21354418 PMC3076511

[B6] Correia C. Lee S. H. Meng X. W. Vincelette N. D. Knorr K. L. Ding H. (2015). Emerging understanding of Bcl-2 biology: Implications for neoplastic progression and treatment. Biochim. Biophys. Acta 1853 (7), 1658–1671. 10.1016/j.bbamcr.2015.03.012 25827952 PMC4429517

[B7] De Marchi E. Bonora M. Giorgi C. Pinton P. (2014a). The mitochondrial permeability transition pore is a dispensable element for mitochondrial calcium efflux. Cell calcium 56 (1), 1–13. 10.1016/j.ceca.2014.03.004 24755650 PMC4074345

[B8] De Marchi E. Bonora M. Giorgi C. Pinton P. (2014b). The mitochondrial permeability transition pore is a dispensable element for mitochondrial calcium efflux. Cell Calcium 56 (1), 1–13. 10.1016/j.ceca.2014.03.004 24755650 PMC4074345

[B9] Dewangan J. Srivastava S. Mishra S. Pandey P. K. Divakar A. Rath S. K. (2018b). Chetomin induces apoptosis in human triple-negative breast cancer cells by promoting calcium overload and mitochondrial dysfunction. Biochem. Biophys. Res. Commun. 495 (2), 1915–1921. 10.1016/j.bbrc.2017.11.199 29208466

[B10] Dewangan J. Srivastava S. Mishra S. Pandey P. K. Divakar A. Rath S. K. (2018a). Chetomin induces apoptosis in human triple-negative breast cancer cells by promoting calcium overload and mitochondrial dysfunction. Biochem. Biophys. Res. Commun. 495 (2), 1915–1921. 10.1016/j.bbrc.2017.11.199 29208466

[B11] Duran I. Lambea J. Maroto P. González-Larriba J. L. Flores L. Granados-Principal S. (2017). Resistance to targeted therapies in renal cancer: The importance of changing the mechanism of action. Target. Oncol. 12 (1), 19–35. 10.1007/s11523-016-0463-4 27844272

[B12] Francis A. P. Ganapathy S. Palla V. R. Murthy P. B. Devasena T. (2015). Future of nano bisdemethoxy curcumin analog: Guaranteeing safer intravenous delivery. Environ. Toxicol. Pharmacol. 39 (1), 467–474. 10.1016/j.etap.2014.12.018 25596481

[B13] Guo H. Zhang D. Li C. Jia L. Liu G. Hao L. (2013). Self-assembled nanoparticles based on galactosylated O-carboxymethyl chitosan-graft-stearic acid conjugates for delivery of doxorubicin. Int. J. Pharm. 458 (1), 31–38. 10.1016/j.ijpharm.2013.10.020 24140544

[B14] Heidegger I. Pircher A. Pichler R. (2019). Targeting the tumor microenvironment in renal cell cancer biology and therapy. Front. Oncol. 9, 490. 10.3389/fonc.2019.00490 31259150 PMC6587703

[B15] Hsieh J. J. Purdue M. P. Signoretti S. Swanton C. Albiges L. Schmidinger M. (2017). Renal cell carcinoma. Nat. Rev. Dis. Prim. 3, 17009. 10.1038/nrdp.2017.9 28276433 PMC5936048

[B16] Hu L. Chen M. Chen X. Zhao C. Fang Z. Wang H. (2020). Chemotherapy-induced pyroptosis is mediated by BAK/BAX-caspase-3-GSDME pathway and inhibited by 2-bromopalmitate. Cell Death Dis. 11 (4), 281. 10.1038/s41419-020-2476-2 32332857 PMC7181755

[B17] Izzo V. Bravo-San Pedro J. M. Sica V. Kroemer G. Galluzzi L. (2016). Mitochondrial permeability transition: New findings and persisting uncertainties. Trends Cell Biol. 26 (9), 655–667. 10.1016/j.tcb.2016.04.006 27161573

[B18] Jan R. Chaudhry G. E. (2019). Understanding apoptosis and apoptotic pathways targeted cancer therapeutics. Adv. Pharm. Bull. 9 (2), 205–218. 10.15171/apb.2019.024 31380246 PMC6664112

[B19] Kang F. Zhang S. Chen D. Tan J. Kuang M. Zhang J. (2021). Biflavonoids from selaginella doederleinii as potential antitumor agents for intervention of non-small cell lung cancer. Molecules 26 (17), 5401. 10.3390/molecules26175401 34500834 PMC8434134

[B20] Kundishora A. Sithole S. Mukanganyama S. (2020). Determination of the cytotoxic effect of different leaf extracts from Parinari curatellifolia (chrysobalanaceae). J. Toxicol. 2020, 8831545. 10.1155/2020/8831545 33178265 PMC7644334

[B21] Lai C. Y. Yu G. S. Xu Y. Wu X. Heng B. L. Xue Y. J. (2017). Engrailed-2 promoter hyper-methylation is associated with its downregulation in clear cell renal cell carcinoma. Oncol. Lett. 14 (6), 6888–6894. 10.3892/ol.2017.7000 29151918 PMC5678243

[B22] Li X. Yin A. Zhang W. Zhao F. Lv J. Lv J. (2018). Jam3 promotes migration and suppresses apoptosis of renal carcinoma cell lines. Int. J. Mol. Med. 42 (5), 2923–2929. 10.3892/ijmm.2018.3854 30226554

[B23] Lin Y. C. Su J. H. Lin S. C. Chang C. C. Hsia T. C. Tung Y. T. (2018). A soft coral-derived compound, 11-dehydrosinulariolide, induces G2/M cell cycle arrest and apoptosis in small cell lung cancer. Mar. Drugs 16 (12), E479. 10.3390/md16120479 PMC6315988 30513611

[B24] Liu H. Yang J. Q. Ma H. M. Wang B. (2015). Analysis of steroidal saponins from Reineckia carnea and their antitumor activities. Traditional Chin. Drug Res. Clin. Pharmacol. 26 (03), 348–351.

[B25] Liu H. Yang J. Q. Xiong L. Wang Z. (2012). Study on chemical constituents and pharmacological activities of carnationgrass. Chin. Tradit. Pat. Med. 34 (09), 1785–1789.

[B26] Ljungberg B. Albiges L. Abu-Ghanem Y. Bedke J. Capitanio U. Dabestani S. (2022). European association of urology guidelines on renal cell carcinoma: The 2022 update. Eur. Urol. 82 (4), 399–410. 10.1016/j.eururo.2022.03.006 35346519

[B27] Lopez J. Tait S. W. (2015). Mitochondrial apoptosis: Killing cancer using the enemy within. Br. J. Cancer 112 (6), 957–962. 10.1038/bjc.2015.85 25742467 PMC4366906

[B28] Ma Q. G. Tang Y. Sang Z. P. Dong J. H. Wei R. R. (2021). Structurally diverse biflavonoids from the fruits of Citrus medica L. var. sarcodactylis Swingle and their hypolipidemic and immunosuppressive activities. Bioorg. Chem. 117, 105450. 10.1016/j.bioorg.2021.105450 34710667

[B29] Montero J. A. Lorda-Diez C. I. Hurle J. M. (2020). Confluence of cellular degradation pathways during interdigital tissue remodeling in embryonic tetrapods. Front. Cell Dev. Biol. 8, 593761. 10.3389/fcell.2020.593761 33195267 PMC7644521

[B30] Patel P. Mendoza A. Robichaux D. J. Wang M. C. Wehrens X. H. T. Karch J. (2021). Inhibition of the anti-apoptotic bcl-2 family by BH3 mimetics sensitize the mitochondrial permeability transition pore through Bax and bak. Front. Cell Dev. Biol. 9, 765973. 10.3389/fcell.2021.765973 34926454 PMC8672142

[B31] Pfeffer C. M. Singh A. T. K. (2018). Apoptosis: A target for anticancer therapy. Int. J. Mol. Sci. 19 (2), E448. 10.3390/ijms19020448 PMC5855670 29393886

[B32] Pullen R. L. Jr. (2021). Renal cell carcinoma, part 1. Nursing 51 (7), 34–40. 10.1097/01.NURSE.0000753972.19135.dc 34156999

[B33] Ren Y. Huang S. S. Wang X. Lou Z. G. Yao X. P. Weng G. B. (2016). Ginkgetin induces apoptosis in 786-O cell line via suppression of JAK2-STAT3 pathway. Iran. J. Basic Med. Sci. 19 (11), 1245–1250. 27917282 PMC5126227

[B34] Schrader A. J. Varga Z. Hegele A. Pfoertner S. Olbert P. Hofmann R. (2006). Second-line strategies for metastatic renal cell carcinoma: Classics and novel approaches. J. Cancer Res. Clin. Oncol. 132 (3), 137–149. 10.1007/s00432-005-0058-4 16308709 PMC12161062

[B35] Shim S. Y. Lee S. G. Lee M. (2018). Biflavonoids isolated from selaginella tamariscina and their anti-inflammatory activities via ERK 1/2 signaling. Molecules 23 (4), E926. 10.3390/molecules23040926 PMC6017943 29673161

[B36] Šileikytė J. Forte M. (2019). The mitochondrial permeability transition in mitochondrial disorders. Oxid. Med. Cell. Longev. 2019, 3403075. 10.1155/2019/3403075 31191798 PMC6525910

[B37] Spadaccino F. Netti G. S. Rocchetti M. T. Castellano G. Stallone G. Ranieri E. (2020). Diagnostic and prognostic markers of renal cell carcinoma. G. Ital. Nefrol. 37 (2), 2020. 32281759

[B38] Sung H. Ferlay J. Siegel R. L. Laversanne M. Soerjomataram I. Jemal A. (2021). Global cancer statistics 2020: GLOBOCAN estimates of incidence and mortality worldwide for 36 cancers in 185 countries. Ca. Cancer J. Clin. 71 (3), 209–249. 10.3322/caac.21660 33538338

[B39] Xiang W. Zhang R. J. Jin G. L. Tian L. Cheng F. Wang J. Z. (2020). RCE-4, a potential anti-cervical cancer drug isolated from Reineckia carnea, induces autophagy via the dual blockade of PI3K and ERK pathways in cervical cancer CaSki cells. Int. J. Mol. Med. 45 (1), 245–254. 10.3892/ijmm.2019.4389 31746346

[B40] Xiang Y. Guo Z. Zhu P. Chen J. Huang Y. (2019). Traditional Chinese medicine as a cancer treatment: Modern perspectives of ancient but advanced science. Cancer Med. 8 (5), 1958–1975. 10.1002/cam4.2108 30945475 PMC6536969

[B41] Xiao S. Mu Z. Q. Cheng C. R. Ding J. (2019). Three new biflavonoids from the branches and leaves of Cephalotaxus oliveri and their antioxidant activity. Nat. Prod. Res. 33 (3), 321–327. 10.1080/14786419.2018.1448817 29544363

[B42] Xie Y. Zhou X. Li J. Yao X. C. Liu W. L. Xu P. S. (2022). Cytotoxic effects of the biflavonoids isolated from Selaginella trichoclada on MCF-7 cells and its potential mechanism. Bioorg. Med. Chem. Lett. 56, 128486. 10.1016/j.bmcl.2021.128486 34875389

[B43] Xing X. F. Jin G. L. Xi W. Chen J. F. Cheng F. Zou K. (2018). Research progress of medicinal reineckea carnea. Her. Med. 37 (10), 1233–1236.

[B44] Xue Y. J. Chen S. N. Chen W. G. Wu G. Q. Liao Y. F. Xu J. B. (2019). Cripto-1 expression in patients with clear cell renal cell carcinoma is associated with poor disease outcome. J. Exp. Clin. Cancer Res. 38 (1), 378. 10.1186/s13046-019-1386-6 31455359 PMC6712621

[B45] Yang J. Q. Wang Y. Yan C. Wang N. N. Hao X. Y. (2010). Chemical constituents from Reineckia carnea kunt. Nat. Prod. Res. Dev. 22 (02), 245–247. 10.16333/j.1001-6880.2010.02.004

[B46] Zhou J. Jiang Y. Y. Chen H. Wu Y. C. Zhang L. (2020). Tanshinone I attenuates the malignant biological properties of ovarian cancer by inducing apoptosis and autophagy via the inactivation of PI3K/AKT/mTOR pathway. Cell Prolif. 53 (2), e12739. 10.1111/cpr.12739 31820522 PMC7046305

[B47] Zhou X. Liu H. Gong X. J. Zhao C. Chen H. G. (2010). A new flavone from reineckea carnea. Chin. Pharm. J. 45 (01), 16–18.

